# CAMK2B Impacts the Proliferation, Invasion, and Migration of Glioma Cells via the Ras/Raf/MEK/ERK Signaling Pathway

**DOI:** 10.32604/or.2025.064300

**Published:** 2025-09-26

**Authors:** Shiyang Zhang, Jingchen Li, Qianxu Jin, Siyu Zhu, Hongshan Yan, Yizheng Wang, Zihan Song, Liqiang Liu

**Affiliations:** 1 Department of Neurosurgery, The Second Hospital of Hebei Medical University, Shijiazhuang, 050000, China; 2 Department of Neurosurgery, The Fourth Hospital of Hebei Medical University, Shijiazhuang, 050000, China; 3 Department of Pain and Rehabilitation, The Fourth Hospital of Hebei Medical University, Shijiazhuang, 050000, China

**Keywords:** Calcium/calmodulin-dependent protein kinase II beta (CAMK2B), glioma, malignant progression, Ras/Raf/MEK/ERK pathway

## Abstract

**Background:**

Glioma is the most common tumor of the central nervous system with a poor prognosis. This study aims to explore the role of calcium/calmodulin-dependent protein kinase IIβ (CAMK2B) in regulating the malignant progression of glioma cells, as well as the molecular mechanisms underlying these malignant behaviors.

**Methods:**

The correlation between CAMK2B expression in gliomas and patient prognosis was analyzed using immunohistochemistry, quantitative reverse transcription polymerase chain reaction (qRT-PCR), and western blot. Furthermore, the study explored the role of CAMK2B in glioma cell proliferation, invasion, and migration using cell counting kit-8 (CCK-8), 5-Ethynyl-2^′^-deoxyuridine (EdU), wound healing, transwell, and *in vivo* tumor xenograft assays.

**Result:**

Patients with high CAMK2B expression exhibited significantly better prognostic outcomes compared to those with low expression levels. Furthermore, CAMK2B expression was significantly lower in glioma tissues and cells compared to both normal brain tissue and human astrocyte cell lines. Notably, overexpression of CAMK2B in glioma cells led to an approximate 40% reduction in proliferative capacity and a 60–70% decrease in invasive and migratory abilities, compared to control glioma cells. These differences were statistically significant at *p* < 0.05. Conversely, knockdown of CAMK2B using siRNA-CAMK2B significantly enhanced the proliferative, invasive, and migratory capabilities of glioma cells in both *in vitro* and *in vivo* settings, enhancing these abilities by 1.5 to 3 times. Notably, these effects were reversed through the application of the Rat Sarcoma viral oncogene homolog (Ras) pathway inhibitor, Salirasib. Western blot analysis revealed that knockdown of CAMK2B led to activation of the Ras/Rapidly Accelerated Fibrosarcoma (Raf)/Mitogen-activated protein kinase kinase (MEK)/Extracellular signal-regulated kinase (ERK) signaling pathway in glioma cell lines, whereas overexpression of CAMK2B resulted in the suppression of this pathway.

**Conclusion:**

CAMK2B inhibits glioma proliferation, invasion, and migration through the Ras/Raf/MEK/ERK signaling pathway.

## 1 Introduction

Gliomas are primary tumors that arise in various regions of the brain and account for approximately 81% of all malignant tumors. Among these, glioblastomas are responsible for 48% of cases and are characterized by their aggressive malignancy [1]. Epidemiological data indicate a higher incidence of glioblastoma in males compared to females, with a greater prevalence observed in individuals over 65 years of age [2]. Notably, the incidence of glioblastoma in the elderly is approximately five times higher than in younger individuals [3]. Surgical resection is the main treatment approach to gliomas; however, complete removal is often challenging due to the infiltrative nature of the tumor, contributing to a suboptimal prognosis. Adjuvant therapies, including postoperative chemotherapy and radiotherapy, can modestly improve prognosis and slightly prolong overall survival [4]. Despite the use of multimodal therapies, tumor recurrence is almost inevitable, and the median survival following diagnosis remains under two years [5]. Temozolomide is currently the standard chemotherapeutic agent used in glioma management; however, resistance to this drug frequently develops, limiting its long-term efficacy [6]. The five-year survival rate for patients with glioblastoma remains poor, ranging from 0.05% to 4.7% [6–8]. Thus, elucidating novel molecular mechanisms underlying glioma proliferation, invasion, and migration is imperative for the development of more effective therapeutic strategies.

Calcium/calmodulin-dependent protein kinase II (CaMKII) is a serine/threonine-specific protein kinase that plays a central role in regulating the intracellular Ca^2+^ signaling pathway [9]. Previous studies have shown that calcium/calmodulin-dependent protein kinase II beta (CAMK2B) plays a significant role in the growth and development of neurons and synaptic plasticity; additionally, it is implicated in various neuropathological processes involving sleep disorders [10], memory disorder, severe intellectual disability [11], mental retardation [12], schizophrenia is, neurodevelopmental disorder [13,14], and Alzheimer’s disease [15]. Analysis of data from The Cancer Genome Atlas (TCGA), Chinese Glioma Genome Atlas (CGGA), Repository for Molecular Brain Neoplasia Data (Rembrandt), and Gene Expression Profiling Interactive Analysis (GEPIA) databases reveals that the expression of CAMK2B is significantly reduced in both low-grade gliomas and glioblastomas compared to normal brain tissue. This finding indicates a potential relationship between reduced CAMK2B levels and low-grade glioma development, as well as its prognostic value in glioma progression. Moreover, CAMK2A, a homolog of CAMK2B, has been reported to significantly suppress glioma proliferation and metastasis induced by miR-3200-3p when it is overexpressed. The interaction between CAMK2A and miR-3200-3p has been demonstrated to regulate the progression of glioma through the Rat Sarcoma viral oncogene homolog (Ras)/Rapidly Accelerated Fibrosarcoma (Raf)/Mitogen-activated protein kinase kinase (MEK)/Extracellular signal-regulated kinase (ERK) signaling pathway [16]. Additionally, CAMK2D, a member of the CAMK2 family, has been shown to form complexes with RNF8 and MAD2, thereby contributing to the regulation of the mitotic checkpoint in glioma cells. This interaction may serve as a mitotic checkpoint signal and represent a potential therapeutic target for glioma treatment [17]. Despite these findings, the molecular mechanism underlying the clinical significance of CAMK2B in glioma remains largely unknown. Consequently, this study aims to elucidate the role of CAMK2B in the pathogenesis of glioma, as well as the signaling pathways it exerts its therapeutic effects, by conducting *in vitro* and *in vivo* experiments.

## 2 Materials and Methods

### 2.1 Bioinformatics Analysis

The GEPIA database (http://gepia.cancer-pku.cn/), an online resource for interactive gene expression profiling, was used to analyze CAMK2B expression across various tumor and normal tissue types. Additionally, RNAseq data for glioma samples were obtained from the TCGA (https://portal.gdc.cancer.gov), CGGA (http://www.cgga.org.cn), and Rembrandt (https://gliovis.bioinfo.cnio.es/) databases, and subsequently curated for downstream analysis. The mRNA transcriptome data were converted into the FPKM format for further analysis using the log_2_(value + 1) method. All prognostic data for patients were obtained from the relevant websites. Additionally, all the information from the databases was accessed in August 2022. The median expression level of CAMK2B was used as the cut-off value to classify glioma patients into high and low-expression groups. The Kaplan-Meier survival analysis was conducted using the “survminer (version 3.3.1)” and “survival (version 0.4.9)” packages in R (version 4.4.1) to investigate the association between CAMK2B expression and patient prognosis.

### 2.2 Cell Culture and Treatment

Human astrocytes (HA) were acquired from ScienCell (Carlsbad, CA, USA). Three human glioma cell lines (U251, U87, and A172) were acquired from Procell Life Science & Technology Co., Ltd. (Wuhan, China). All cells were identified by Short Tandem Repeat (STR) and there was no risk of mycoplasma infection. The Roswell Park Memorial Institute (RPMI)-1640 medium (Gibco™, 11875093, Thermo Fisher Scientific, Grand Island, NY, USA) was used to culture the U251 cells. The U87 cells were cultured in Minimum Essential Medium (MEM, Gibco™, 11090081, Thermo Fisher Scientific), while the A172 cells were cultured in Dulbecco’s modified Eagle’s medium (DMEM, Gibco™, 10564011, Thermo Fisher Scientific). The HA cells were cultured in an Astrocyte medium (AM, Sciencell™, #1801, Carlsbad, CA, USA). All culture media were supplemented with 10% fetal bovine serum (FBS, Gibco™, 10099141, Thermo Fisher Scientific) and 1% penicillin-streptomycin. Cell culturing was conducted in a cell incubator at 37°C and 5% carbon dioxide. The Ras pathway inhibitor salirasib (SML1166, Sigma-Aldrich Corporation, St. Louis, MO, USA) was diluted to 150 μM with DMSO, co-cultured with the cells scheduled for the experiment for 48 h, and then the subsequent experiments could be conducted.

### 2.3 Cell Transfection

The CAMK2B−coding sequence was synthesized and cloned into the pcDNA 3.1 vector by Hanbio Biotechnology Co., Ltd. (Shanghai, China) to construct a CAMK2B overexpression plasmid (pcCAMK2B). The blank pcDNA3.1 plasmid was used as a negative control (NC) plasmid. The siRNA for silencing CAMK2B was designed and synthesized by Thermo Fisher Scientific. The targeting sequence for siCAMK2B was CAGAAGAATGGTACAAATCCAAG. Plasmids (8 μg/10^6^ cells) or small interfering RNA (siRNA) (60 pmol/10^6^ cells) were transfected in glioma cells using Lipofectamine 3000 (L3000015, Thermo Fisher Scientific), according to the product instructions. After 48–72 h, all cells were collected and used in the follow-up experiments.

### 2.4 Clinical Samples

A total of 79 glioma tissue samples and 4 non-tumorous brain tissue samples (excised from patients undergoing brain trauma surgery) were attained from the Second Hospital of Hebei Medical University on 15 December 2021. The surgical collection of tissue samples was conducted between 24 September 2014, and 09 March 2019. Patient follow-up for survival analysis was completed on 20 December 2021. The remaining samples were stored in 10% paraformaldehyde for immunohistochemistry (IHC) experiments. This research was approved by the ethics committee of the Second Hospital of Hebei Medical University (Approval No. 2021-R494). Informed and written consent was obtained from either the patient or their guardian following the guidelines provided by the ethics committee.

### 2.5 Quantitative Reverse Transcription-Polymerase Chain Reaction (qRT-PCR)

Total RNA was extracted from untreated and transfected U251 and U87 glioma cells using Trizol reagent (15596026CN, Thermo Fisher Scientific, Waltham, MA, USA) according to the manufacturer’s instructions. After RNA quantification, cDNA was synthesized using a reverse transcription kit, HiScript^®^ III RT SuperMix for qPCR (+gDNA wiper) (R232-01, Vazyme, Nanjing, China), according to the manufacturer’s instructions. With GAPDH as the standardized internal reference, AceQ Universal SYBR qPCR Master Mix (Vazyme, Nanjing, China) was used to conduct the PCR procedure. The qPCR thermal cycling conditions were as follows: an initial denaturation step at 95°C for 10 min (1 cycle), followed by 40 amplification cycles consisting of denaturation at 95°C for 5 s, annealing at 60°C for 30 s, and extension at 72°C for 30 s. The primer sequences (Species: Human) of CAMK2B were as follows: F, 5^′^-GCAAAGAGGCGTATGGCAAG-3^′^; R, 5^′^-GACGGGAAGTCATAGGCACC-3^′^. The primer sequences (Species: Human) of GAPDH were as follows: F, 5^′^-GGAGCGAGATCCCTCCAAAAT-3^′^; R, 5^′^-GGCTGTTGTCATACTTCTCATGG-3^′^. The relative gene expression was determined using the 2^−ΔΔCt^ method, and GAPDH was used as the reference gene for normalization.

### 2.6 Immunohistochemistry (IHC)

The tissue samples were fixed in formalin, embedded in paraffin, and subsequently sectioned for histological analysis. The tissue sections were incubated at 60°C for 30 min, immersed in xylene, and washed using graded ethanol to remove paraffin. The tissue sections were subjected to antigen retrieval by immersion in 2% citric acid buffer under high temperature and pressure conditions for 25 min, followed by treatment with endogenous peroxidase to inhibit non-specific background staining. Following antigen retrieval, the tissue sections were blocked with 10% goat serum. The sections were then incubated overnight at 4°C with primary antibodies (11533-1-AP, Proteintech, Rosemont, IL, USA) diluted at a 1:100 ratio. After three washes, the sections were then incubated with biotin-labeled goat anti-Rabbit IgG secondary antibody (sp-9001, Zhongshan Golden Bridge Bio-technology, Beijing, China), followed by incubation with streptavidin-biotin complex containing horseradish peroxidase (sp-9001, Zhongshan Golden Bridge Bio-technology, Beijing, China) for 45 min. Immunoreactivity was visualized using 3,3^′^-Diaminobenzidine Tetrahydrochloride at a dilution ratio of 1:150. (DAB; sp-9001, Zhongshan Golden Bridge Bio-technology, Beijing, China). Finally, the stained sections were examined and imaged using an optical microscope (DM IL LED, Leica, Wetzlar, Germany).

### 2.7 Western Blot

Total protein extraction was conducted from the cells using RIPA buffer (R0010, Solarbio, Beijing, China) supplemented with protease and phosphatase inhibitors to prevent protein degradation and dephosphorylation. Subsequently, the concentration of total protein was quantified using the bicinchoninic acid (BCA; RW0201, Hebei Ruipate Biotechnology Co., Ltd., Hebei, China) method. Equal amounts of protein were separated by 10% SDS-PAGE and transferred onto Polyvinylidene Fluoride Membrane (PVDF) membranes (IPVH00010, Immobilon, Darmstadt, Germany). The membranes were blocked with 5% bovine serum albumin (BSA) (SB-PR079, ShareBio, Shanghai, China) at room temperature for 40 min, and incubated overnight at 4°C with primary antibodies against CAMK2B (1:1000) (11533-1-AP, Proteintech, Rosemont, IL, USA), GAPDH (1:7500) (10494-1-AP, Proteintec), Ras (1:1000) (12063-1-AP, Proteintech), Raf1 (1:1000) (26863-1-AP, Proteintech). The following day, the membranes were washed three times with TBST (pH 7.4, concentration 5%) (N8770, Solarbio, Beijing, China) (each 5 min) and then incubated with a preabsorbed goat anti-rabbit IgG H&L secondary antibody at a dilution ratio of 1:2000 (A23920, Abbkine, Wuhan, China) (1:10,000). Protein bands were detected using the Odyssey infrared scanner (Odyssey DLx, LICOR, Lincoln, NE, USA), and the relative expression of CAMK2B was evaluated using ImageJ software (v1.54d, National Institutes of Health, Bethesda, MD, USA).

### 2.8 5-Ethynyl-2^′^-Deoxyuridine (EdU) Assay

A total of 5000 cells were inoculated into each well of a 96-well plate and cultured overnight. Cell proliferation was assessed using the 488 Click-iT EdU Cell Proliferation Kit (C0071S, Beyotime, Shanghai, China) according to the manufacturer’s instructions. The following day, the cells were incubated with 10 μM EdU for 2 h. Cells were fixed, permeabilized, stained, and mounted according to the manufacturer’s instructions. Nuclear counterstaining was performed using Hoechst 33342 (SB-C6043, ShareBio, Shanghai, China). The results were observed and images were captured using a fluorescence microscope (DM IL LED, Leica, Wetzlar, Germany), with ImageJ software used to conduct quantitative analysis.

### 2.9 Cell Counting Kit-8 (CCK-8) Assay

A total of 5000 cells were inoculated into a 96-well plate and cultured for 24, 48, 72, and 96 h. Subsequently, 10 μL CCK-8 (SB-CCK8, ShareBio, Shanghai, China) (5 mg/mL) was added to each well and the cells were incubated for another 2 h under conditions of 37°C and 5% carbon dioxide. The OD value was assessed using a microplate reader (Multiskan FC, Thermo Fisher Scientific, Waltham, MA, USA), with the absorbance determined at 450 nm.

### 2.10 Transwell Assay

Treated cells (20,000 cells per well) were inoculated into the upper chamber of a Transwell insert containing serum-free medium. The bottom of the upper compartment was pre-coated with Matrigel, while the lower compartment was filled with a complete medium. Cells were cultured at 37°C, with 5% carbon dioxide for 24 h. Subsequently, the migrated cells were fixed with 4% paraformaldehyde for 30 min and then stained with 1% crystal violet. The invasive cells were then visualized with an optical microscope (DMIL LED, Leica).

### 2.11 Wound Healing Assay

The treated glioma cells were inoculated into 6-well plates. Once the cells reached the desired confluence and formed a dense monolayer, a scratch was introduced using a pipette tip. The cells were then cultured in a serum-free medium to assess migration. Images of the scratch area were captured with an optical microscope (DMIL LED, Leica) at 0 and 24 h in the same region. The wound healing area was analyzed with ImageJ software.

### 2.12 Tumor Xenotransplantation Assay

Four-week-old male BALB/c-nu nude mice were purchased from Beijing Huafukang Biotechnology Co., Ltd. (Beijing), with an initial body weight of 15–20 g per mouse. The mice were randomly divided into 2 groups, with 6 mice in each group, and housed in an environment maintained at 20°C–22°C, 65%–79% humidity, and a 12-h light-dark cycle, with free access to food and water. After 1 week of acclimatization to the controlled environment and regular feeding, U251 cells transfected with siCAMK2B and siNC were cultured to 90% confluence, prepared into a cell suspension at a concentration of 5 × 10^6^ cells/mL, and subcutaneously injected into the right flanks of the mice. Visible tumors typically formed subcutaneously within 3 days post-injection, and then mouse weights and tumor dimensions were measured every 7 days for 4 weeks. Tumor volume was calculated using the equation of *V* = ½ × *length* × *width*^2^. At the end of the observation period, the mice were anesthetized and euthanized, and the tumors were excised, weighed, and photographed for further analysis. This experimental procedure was approved by the Ethics Committee of the Second Hospital of Hebei Medical University and performed following the established protocols (Approval No. 2021-AE047).

### 2.13 Statistical Analysis

All experiments were conducted in triplicate. Quantitative data were expressed as mean ± standard deviation (SD), with the Student’s *t*-test used to compare data between two groups. The Kaplan-Meier method with a log-rank test was used to plot the survival curve for glioma patients based on the expression level of CAMK2B. The GraphPad Prism 9.0 software (v121, GraphPad Software, San Diego, CA, USA) was used to process all the data for statistical analysis. A *p*-value <0.05 was considered to be statistically significant.

## 3 Result

### 3.1 Patients with High CAMK2B Expression Exhibit a Superior Prognosis

**Figure 1:**
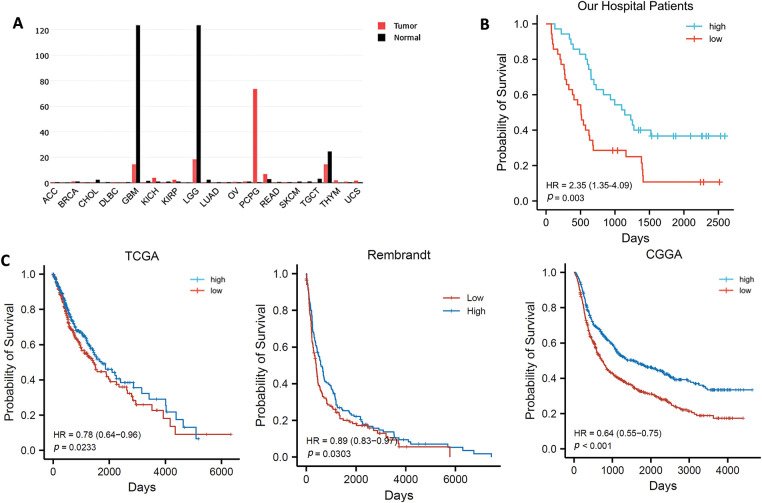
CAMK2B expression in pan carcinoma and survival analysis of CAMK2B in patients with glioma. (**A**) CAMK2B expression in pan carcinoma. (**B**) Survival analysis of patients from the Second Hospital of Hebei Medical University. (**C**) Survival analysis of patients from TCGA, CCGA, and Rambrandt databases

Based on transcriptome data obtained from the GEPIA database, we found that the expression level of CAMK2B was lower in glioma tissues compared to normal brain tissues (Fig. 1A). Immunohistochemical analysis of 70 patients in our hospital showed that the patients with high levels of CAMK2B expression exhibited better prognostic outcomes compared to those with low levels of CAMK2B (Fig. 1B). Analysis results from the TCGA, CCGA, and Rambrandt databases exhibited consistent findings (Fig. 1C) with those obtained using the GEPIA database (Fig. 1A).

### 3.2 Glioma Cells and Tissues Exhibit Low Levels of CAMK2B

**Figure 2:**
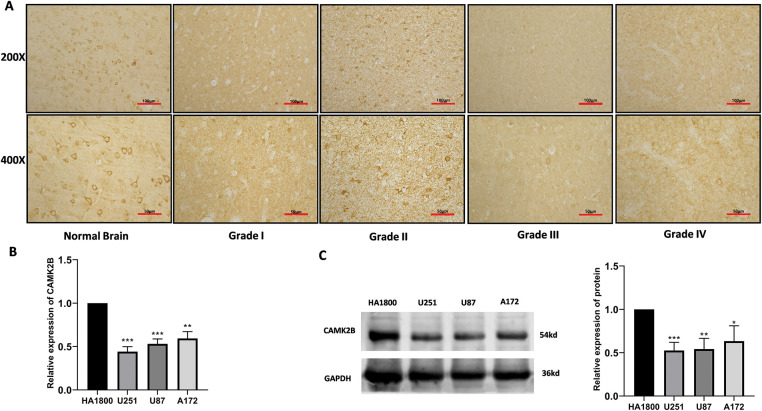
CAMK2B expression in tissues and cells. (**A**) Immunohistochemical results showed that compared with normal brain tissue, CAMK2B expression level in glioma decreased with the increase of glioma grade. (**B**,**C**) qRT-PCR and Western Blot results show that CAMK2B is significantly low expressed in three glioma cells (U251, U87, A172) compared with human astrocytes (HA). *p < 0.05; **p < 0.01; ***p < 0.001

We analyzed CAMK2B expression in normal brain tissue and grade I-IV glioma tissue using the IHC technique. The results revealed that CAMK2B expression levels were decreased in tissues of glioma at high grades (Fig. 2A). Additionally, the mRNA levels quantified using the qRT-PCR and protein levels quantified using the Western blot (Fig. 2B,C) yielded similar results, indicating that CAMK2B expression was lower in three glioma cells compared to the astrocytes.

### 3.3 Activation of CAMK2B Attenuated Glioma Cell Proliferation

**Figure 3:**
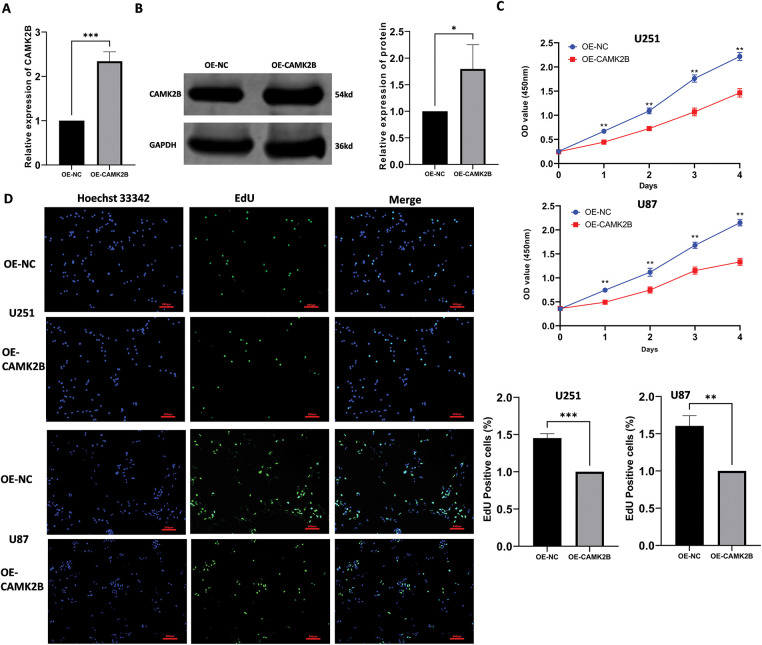
Stimulation of CAMK2B inhibits the proliferation of glioma cells. (**A**,**B**) Detection of CAMK2B expression in U251 cells after plasmid transfection by qRT-PCR and Western Blot. (**C**) CCK-8 assay reveals the effect of CAMK2B on U251 and U87 glioma cell viability. (**D**) EdU assay reveals the effect of CAMK2B on U251 and U87 glioma cell proliferation. *p < 0.05; **p < 0.01; ***p < 0.001

To examine the effects of CAMK2B activation on the proliferation of glioma cells, we overexpressed CAMK2B through the transfection of plasmid into U251 and U87 cells, with transfection of empty plasmids serving as a blank control. The expression levels of CAMK2B in transfected glioma cells (U251) were assessed using qRT-PCR and Western blot (Fig. 3A,B). The expression levels of CAMK2B were significantly upregulated in U251 cells. Additionally, we used CCK-8 and EdU assays to assess the effect of CAMK2B overexpression on the population of U251 and U87 cells; notably, the CCK-8 assay can be used to examine cell viability, which is indicative of cell proliferation. The glioma cells were categorized into two groups: the CAMK2B overexpression group (group OE-CAMK2B) and the blank control group transfected with empty plasmids (group OE-NC). The procedure for assessing cell proliferation involved the following: A total of 5000 cells per well were inoculated into 96-well plates, followed by plasmid transfection, then the OD at 450 nm was measured every four days to assess the proliferation of cells in each group. The results revealed that overexpression of CAMK2B reduced the viability of U251 and U87 cell lines, thereby inhibiting their proliferative capacity (Fig. 3C). These findings were validated through the results from the EdU assay, which revealed that the CAMK2B overexpression group had a lower proportion of proliferating cells compared to cells transfected with the control vectors (Fig. 3D).

### 3.4 Stimulation of CAMK2B Prevented the Invasion and Migration of Glioma Cells

**Figure 4:**
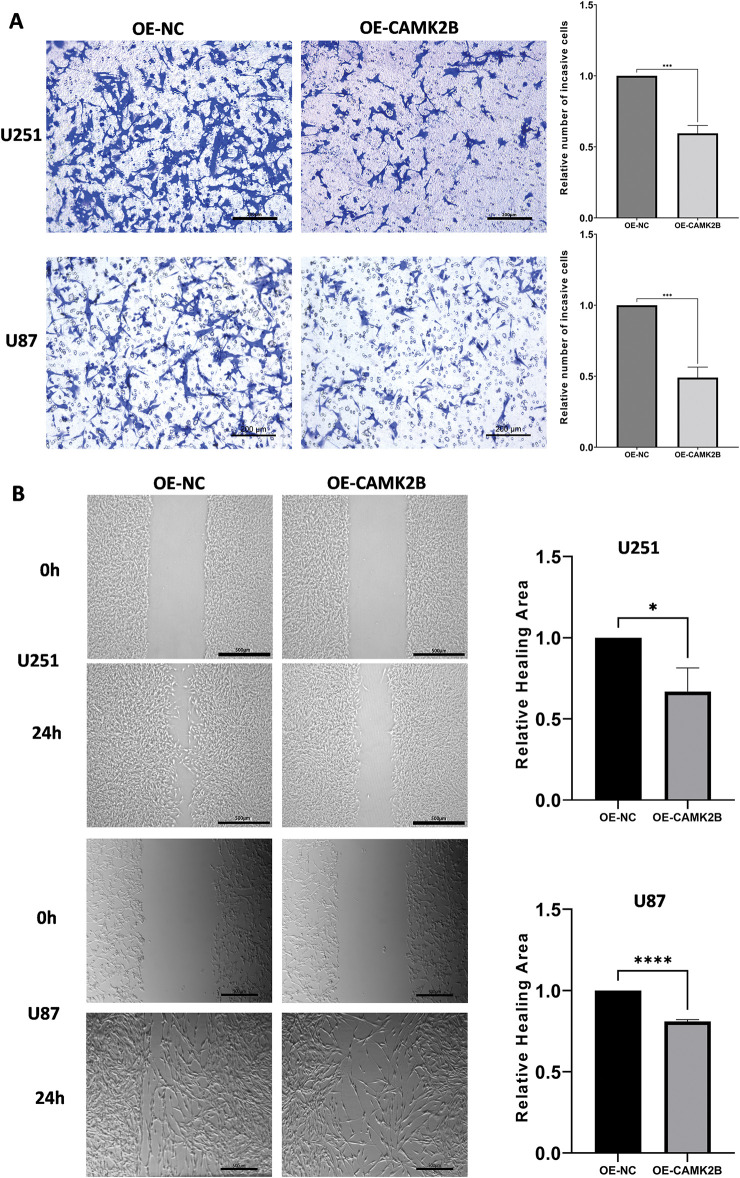
Activating CAMK2B inhibits the invasion and migration of glioma cells. (**A**) The Transwell assay displayed that activated CAMK2B reduced the invasion of U251 and U87 glioma cells. (**B**) The wound healing assay displayed that activated CAMK2B reduced the healing area of U251 and U87 glioma cells. *p < 0.05; ***p < 0.001; ****p < 0.0001

We used Transwell and wound healing assays to assess the impact of activated CAMK2B on the invasive and migrative activity of glioma cells. The U251 and U87 cells transfected with the overexpression plasmid were used for the assays. The U251 and U87 cells transfected with CAMK2B or control plasmids were seeded into the Transwell cell at 0 h, followed by fixation and staining at 24 h. The number of cells migrating through the pores in the OE-CAMK2B group was significantly lower compared to the OE-NC group (Fig. 4A). In the wound healing assay, the healing areas of U251 and U87 cells with overexpression of CAMK2B were smaller compared to those of the cells transfected with the control plasmid (Fig. 4B). These findings indicated that overexpression of CAMK2B prevents the invasion and migration of glioma cells.

### 3.5 Knockdown of CAMK2B Promoted Glioma Proliferation In Vivo

**Figure 5:**
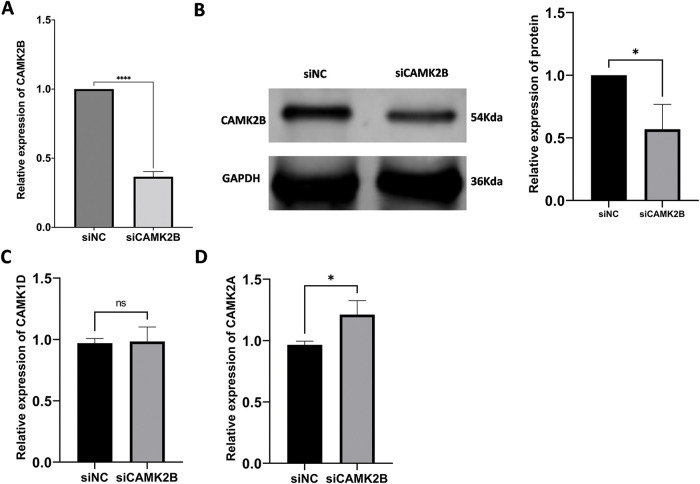

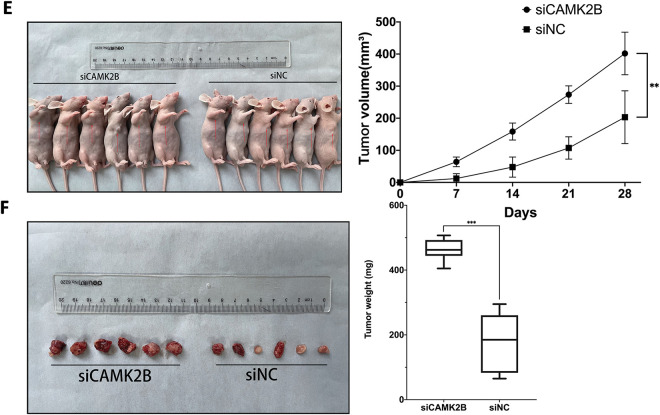
Silencing CAMK2B can promote the proliferation of glioma cells *in vivo*. (**A**,**B**) Detection of CAMK2B expression after siCAMK2B transfection by qRT-PCR and Western Blot. (**C**,**D**) Detection of CAMK1D and CAMK2A expression after siCAMK2B transfection by qRT-PCR. (**E**,**F**) Tumor volume and weight *in vivo* were significantly increased after subcutaneous injection of U251 cells of siCAMK2B into nude mice. *p < 0.05; **p < 0.01; ***p < 0.001; ****p < 0.0001; “ns” stands for no statistical significance

Knockdown of CAMK2B was achieved by transfecting a siRNA plasmid targeting CAMK2B into the glioma cell lines. The expression level of CAMK2B was quantified using the qRT-PCR and Western blot techniques, while the expression of CAMK1D and CAMK2A was quantified using only the qRT-PCR (Fig. 5A–D). The results revealed that the expression level of CAMK2B was downregulated in glioma cells by about 40%. The cells obtained from the siCAMK2B and siNC groups were injected into the nude mice through the armpit. Subsequently, the length, width, and height of the tumor were measured at 7, 14, 21, and 28 days after injection to determine the volume of the tumor. The nude mice were then euthanized on day 28, and the tumor was excised and weighed. Quantitative analysis on day 28 revealed that the tumor volumes were significantly higher in mice subjected to siCAMK2B injection (401.9 ± 66.30 mm^3^, *n* = 6) compared to mice subjected to siCAMK2B injection (203.3 ± 82.23 mm^3^, *n* = 6). Furthermore, the tumor weight of mice injected with siCAMK2B was 463.7 ± 34.64 mg (*n* = 6), which was significantly greater compared to the 178.0 ± 91.93 mg (*n* = 6) in mice injected with siCAMK2B (Fig. 5E,F). These findings demonstrated that the knockdown of CAMK2B promoted glioma proliferation and growth *in vivo*.

### 3.6 Regulation of CAMK2B Expression Levels Mediates Ras/Raf/MEK/ERK Signaling Pathway

**Figure 6:**
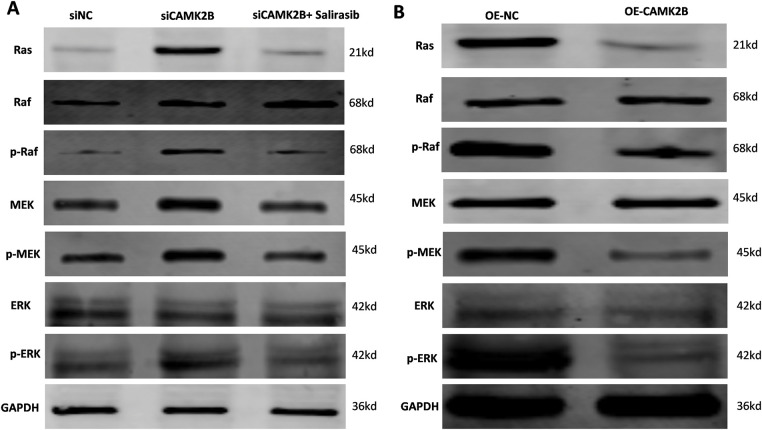
CAMK2B expression affects Ras/Raf/MEK/ERK signal pathway. (**A**) The Ras, p-Raf, p-MEK, and p-ERK were increased after the knock-down of CAMK2B; nevertheless, the Ras inhibitor Salirasib can reverse this change. (**B**) The Ras, p-Raf, p-MEK, and p-ERK were decreased after activating CAMK2B

We regulated the expression of CAMK2B in U251 cells, then used Western blot to determine the protein levels in the Ras/Raf/MEK/ERK signaling pathway. Knockdown of CAMK2B with siRNA resulted in an increased expression level of Ras, p-Raf, p-MEK, and p-ERK; these effects were inhibited by the Ras inhibitor Salirasib, indicating that Salirasib can inhibit the proliferation of glioma cells both *in vivo* and *in vitro* [18,19] (Fig. 6A). Conversely, overexpression of CAMK2B reduced the expression levels of Ras, p-Raf, p-MEK, and p-ERK in U251 cells (Fig. 6B).

### 3.7 Silencing CAMK2B Accelerated the Proliferation, Invasion, and Migration of Glioma Cells through the Ras/Raf/MEK/ERK Signaling Pathway

**Figure 7:**
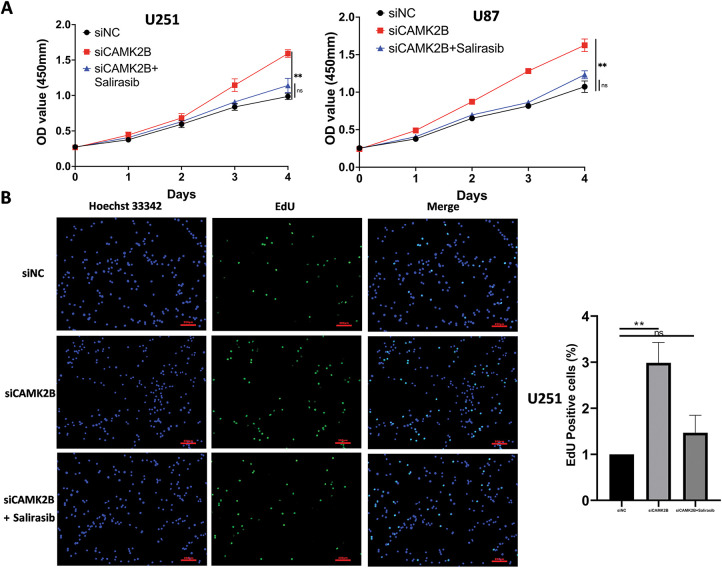

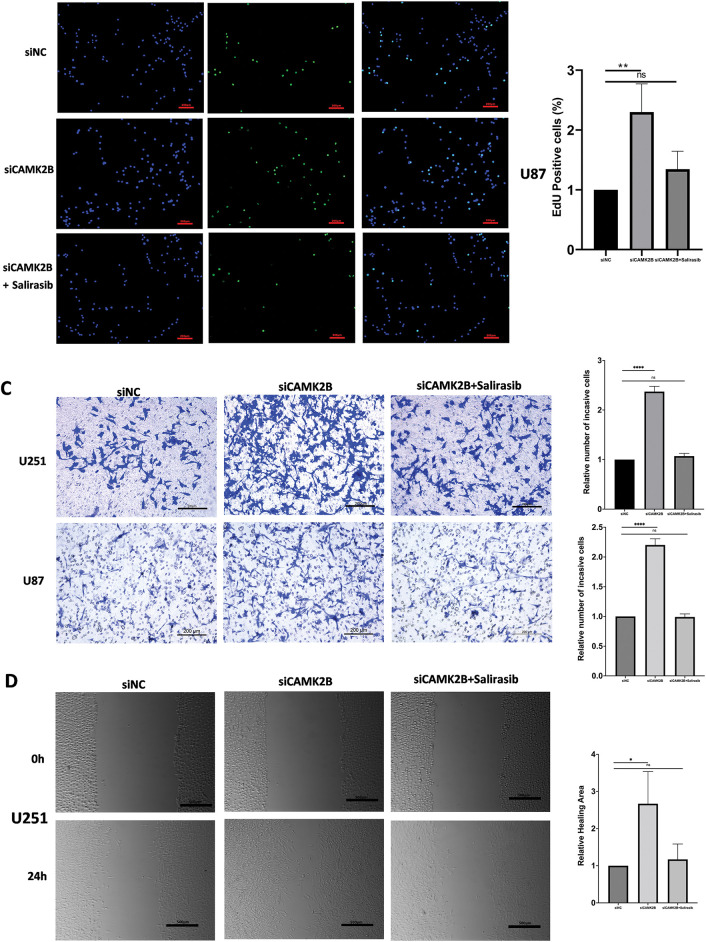

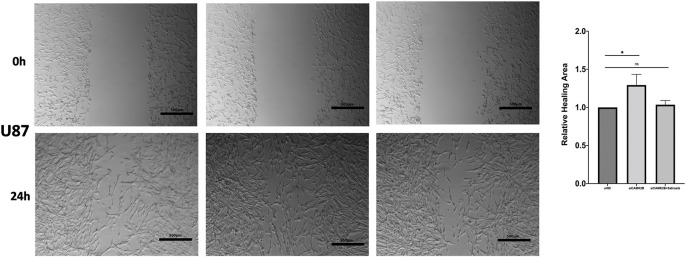
Silencing CAMK2B can promote the proliferation, invasion, and migration of glioma cells, while the Ras inhibitor Salirasib can reverse this effect. (**A**) CCK8 assay points out that silencing CAMK2B can enhance the viability of U251 and U87 glioma cells, and the Ras inhibitor Salirasib can reverse this result. (**B**) EdU assay displayed that silencing CAMK2B could enhance the proliferation of U251 and U87 glioma cells; simultaneously, the Ras inhibitor Salirasib could reverse this result. (**C**) Transwell assay shows that knockdown of CAMK2B can improve the invasive ability of U251 and U87 glioma cells; concurrently, the Ras inhibitor Salirasib can reverse this trend. (**D**) Wound healing assay indicated that the knockdown of CAMK2B can raise the migration ability of U251 and U87 glioma cells, besides the Ras inhibitor Salirasib can reverse this phenomenon. *p < 0.05; **p < 0.01; ****p < 0.0001; “ns” stands for no statistical significance

We conducted *in vitro* experiments using the U87 and U251 glioma cells. Each of the cell lines was divided into three groups, comprising a scrambled control group (siNC), a siCAMK2B group (siCAMK2B), and a siCAMK2B + Salirasib group (siCAMK2B + Salirasib). The CCK8 assay results showed that compared with the control group, the cell viability of the siCAMK2B group was significantly increased on days 3 and 4. However, these effects were reversed in the siCAMK2B + Salirasib group following the addition of the Ras inhibitor Salirasib. Comparable results were observed with the EdU assay, specifically, the siCAMK2B group exhibited increased cell proliferation ability, which was reversed by the Ras inhibitor Salirasib. Knocking down CAMK2B resulted in about a 1.5-fold increase in cell proliferation ability compared to the control group. (Fig. 7A,B). Transwell experiment results showed that compared to the control group, the siCAMK2B group exhibited a significantly increased number of cells penetrating the Matrigel at 24 h, approximately 1.2-fold that of the control group. However, the addition of the Ras inhibitor Salirasib reversed this phenomenon. Consequently, we hypothesize that CAMK2B possesses the ability to regulate the invasive capacity of the glioma cells (Fig. 7C). The wound healing assays showed that compared to the control group, the siCAMK2B group exhibited greater cell migration at 24 h, with its migratory effects greater in the U251 cells compared to the U87 cells. Notably, these effects observed through the wound healing assay were reversed by the Ras inhibitor Salirasib (Fig. 7D). These findings indicate that CAMK2B plays a significant role in the hyperplasia, invasion, and migration of glioma cells through the Ras/Raf/MEK/ERK signaling pathway.

## 4 Discussion

In a previous study, we demonstrated that CAMK1D exerts a significant influence on glioma cell invasion through the phosphatidylinositol-3-kinase/protein kinase B/mammalian target of rapamycin (PI3K/AKT/mTOR) signaling pathway [20]. Consequently, this study investigated whether CAMK2B (a member of the CAMK family) exerts similar impacts on glioma invasion and migration. Research has implicated CAMK2B in the formation of hippocampal neurons in rats [21]. Additionally, CAMK2B can regulate the H1F-1α signaling pathway, thereby safeguarding neurons from apoptosis triggered by homocysteine [22]. Both the Ca^2+^-dependent activity and CaMK2 autonomous activity are essential for cellular survival. In mice, the absence of CAMK2B is fatal [23]. Research has demonstrated that locomotion in mice requires the activation of CAMK2B, which is mediated by calcium/calcium-binding proteins. Notably, the absence of CAMK2B can result in motor impairments in mice [24]. These observations demonstrate that CAMK2B plays a pivotal role in the development and function of the nervous system. Furthermore, CAMK2B contributes to the growth of various tumor types, including papillary renal cell carcinoma, human neuroblastoma, and breast cancer [25]. Additionally, CAMK2B is present in epithelial-mesenchymal transition (EMT) [26].Currently, emerging technologies have been applied to improve cancer diagnosis. For example, liquid biopsy, emerging sequencing technologies, and methylation detection provide new approaches for exploring the role of CAMK2B in glioma and hold significant potential as gene diagnostic tools [27–29]. Notably, liquid biopsy can detect biomarkers associated with CAMK2B; novel sequencing technologies facilitate in-depth analysis of mutations and expression changes in the CAMK2B and related genes; methylation detection facilitates the identification of the impact of the methylation status of the CAMK2B gene promoter region on its expression. Although each of these approaches has contributed to advances in glioma research, further investigation and validation are required to enhance the application of CAMK2B-based gene diagnostics for glioma [30–32].

The Ras/Raf/MEK/ERK (MAPK) signaling pathway is highly responsive and plays a significant role in the pathogenesis of various tumor types. The activation of this pathway is often attributed to oncogenic mutations in key regulatory genes such as the KRAS, NRAS, and BRAF [33]. The accumulation of these genetic alterations plays a pivotal role in tumorigenesis [34,35]. These mutations potentially disrupt physiological cell proliferation, differentiation, and apoptosis [36]. The MAPK signaling transduction is one of the primary pathways in cancer biology, with its disruption implicated in various human pathological studies; specifically, its activation accounts for over 40% of human cancer cases [37,38]. The MAPK signaling has been implicated in the pathogenesis of various cancers, including colon, pancreatic, lung, gastric, and breast cancers [39]. The Ras/Raf/MEK/ERK signaling pathway plays a pivotal role in the apoptotic, autophagic, proliferative, invasive, and migratory processes in glioma [40,41]. The Ras/Raf/MEK/ERK signaling pathway plays a pivotal role in the pathogenesis of glioma. Our findings reveal that modulating the expression level of CAMK2B triggers the activation of the Ras/Raf/MEK/ERK signaling pathway. Salirasib is a widely recognized inhibitor of the Ras pathway, with the ability to suppress the expression of Ras [18,19]. Our findings revealed that Salirasib effectively inhibited the Ras pathway, further corroborating the interaction between CAMK2B and the Ras/Raf/MEK/ERK signaling cascade. Furthermore, based on the KEGG database, we discovered that CAMK2B can exert an influence on the growth and proliferation of glioma cells via the PI3K/AKT/mTOR signaling pathway. Existing research reveals an intricate relationship between the Ras/Raf/MEK/ERK and PI3K/AKT/mTOR signaling pathways. For instance, agonists implicated in Ras-ERK activation exhibit partial overlap with PI3K/mTORC1 signaling [42]. PI3K, a primary effector of Ras, plays a pivotal role in the regulation of key cellular processes, including cell viability, therapeutic resistance, and angiogenesis, during oncogenic Ras activation [43]. Both the Ras and PI3K signaling pathways are key contributors in the regulation of cellular processes such as apoptosis, growth, differentiation, metabolism, as well as the expression of key genes [44,45]. In a previous study, we established that CAMK1D, a member of the CAMK family, influences the malignant progression of glioma via the PI3K signaling pathway [20]. Therefore, we hypothesize that CAMK2B may modulate glioma progression through the PI3K/AKT/mTOR signaling pathway. This mechanistic interaction will serve as a central focus of our future investigations into the functional role of CAMK2B. Additionally, we explored the relationship among CAMK2B, CAMD, and CAMA. The PCR results revealed that suppressing the expression of CAMK2B in the U251 glioma cell line did not significantly alter the expression patterns of CAMK1D. However, the expression level of CAMK2A was slightly upregulated. Some studies have revealed that CAMK2A and CAMK2B may be closely linked, indicating that they can jointly influence the development of certain pathological conditions. Following the reduced expression level of CAMK2B, a compensatory increase in CAMK2A expression can counteract this decline, exemplified by the impact of CAMK2A and CAMK2B deletion on brain neurodevelopment, ultimately leading to intellectual disability [11,12,46]. This viewpoint represents a promising direction for future investigations aimed at elucidating the role of CAMK2B in glioma pathogenesis.

Our study into CAMK2B as a potential therapeutic target for glioma has revealed that therapeutic strategies involving RNA-based therapies, chemical inhibitory compounds, and indirect targeting using MAPK kinase inhibitors possess distinct benefits and limitations. The RNA interference (RNAi) technology can be used to synthesize small interfering RNAs (siRNAs) [47,48], which are effective in recognizing and binding to the messenger ribonucleic acid (mRNA) sequence of CAMK2B. By leveraging intracellular ribozyme activity, CAMK2B mRNA is selectively degraded, effectively inhibiting its expression. This method exhibits high sequence specificity, enabling precise regulation of the target gene while minimizing off-target effects on the expression of normal genes, thus reducing potential side effects. However, a major obstacle lies in the delivery of siRNAs into glioma cells safely and efficiently, as well as ensuring their stability *in vivo* remains a key issue that urgently needs to be resolved [49,50]. Concerning chemical inhibitory compounds, high-throughput screening methodologies hold promise for identifying small-molecule candidates capable of binding with high affinity to the active site of the CAMK2B protein, thereby facilitating targeted inhibition of its activity. By blocking its kinase activity, the key role of CAMK2B in the proliferation, migration, and invasion of glioma cells can be inhibited. Chemical inhibitors exhibit benefits including ease of synthesis, superior stability, and oral availability, which are conducive to clinical applications. However, during the research and development process, it is crucial to ensure the specificity of the candidate compounds for CAMK2B to minimize non-specific inhibition of other kinases, which can cause adverse reactions [51]. Given that CAMK2B promotes the progression of glioma through the activation of the MAPK kinase signaling pathway, indirectly targeting CAMK2B using MAPK kinase inhibitors is a potential therapeutic strategy for glioma. Notably, these inhibitors can block the signal cascade and inhibit the pro-cancer signals transmitted from CAMK2B to tumor cells. This approach leverages the established clinical use of MAPK kinase inhibitors in the treatment of certain cancers, providing a solid foundation for potential therapeutic applications targeting CAMK2B-related pathways [38,52]. However, long-term use of these inhibitors is prone to resistance; additionally, these inhibitors exhibit significant differences in sensitivity among patients. Therefore, accurately screening patient subpopulations that are most likely to benefit from such targeted therapies remains a significant challenge in clinical practice.

In this study, we preliminarily verified the relationship between CAMK2B and the malignant progression of glioma, and demonstrated through a series of experiments that this relationship is established via the Ras/Raf/MEK/ERK signaling pathway. However, our research still has some limitations. CAMK2B can alter the activity of the pathway, but the factors that regulate CAMK2B activity remain uninvestigated. As a Ca^2+^-dependent factor, changes in CAMK2B activity may be mediated by Ca^2+^. Therefore, our future research will focus on this causal relationship to further improve the understanding of the mechanism by which CAMK2B influences the malignant progression of glioma.

In summary, we have demonstrated that the expression level of CAMK2B in gliomas is significantly lower compared to that in normal brain tissues. Additionally, we have revealed that CAMK2B levels exert a profound influence on the proliferative, invasive, and migratory capabilities of glioma cells both *in vitro* and *in vivo*. We have also established that CAMK2B exerts its influence through the Ras/Raf/MEK/ERK signaling pathway, thereby presenting novel therapeutic targets and avenues for future research in the diagnosis and treatment of glioma.

## 5 Conclusion

Notably, CAMK2B exerts its effects on the biological process of glioma by activating the Ras/Raf/MEK/ERK signaling pathway. Specifically, CAMK2B is downregulated in glioma, which indicates that it may serve as a potential prognostic marker and therapeutic target. Furthermore, Ca^2+^, as a potential upstream regulator of CAMK2B, may provide valuable avenues for future investigations into the role of CAMK2B in the malignant progression of glioma.

## Data Availability

The datasets used and/or analysed during the current study are available from the corresponding author on reasonable request. All data datasets generated and/or analysed during the current study are available in the following repository (http://gepia.cancer-pku.cn/, https://tcga-data.nci.nih.gov/tcga/, http://www.cgga.org.cn, https://gliovis.bioinfo.cnio.es/, accessed on 30 July 2025).
